# Targeted Enrichment for Pathogen Detection and Characterization in Three Felid Species

**DOI:** 10.1128/JCM.01463-16

**Published:** 2017-05-23

**Authors:** Justin S. Lee, Ryan S. Mackie, Thomas Harrison, Basir Shariat, Trey Kind, Timo Kehl, Martin Löchelt, Christina Boucher, Sue VandeWoude

**Affiliations:** a Department of Microbiology, Immunology, and Pathology, Colorado State University, Fort Collins, Colorado, USA; b Department of Computer Science, Colorado State University, Fort Collins, Colorado, USA; c German Cancer Research Center, Research Program in Infection, Inflammation and Cancer, Heidelberg, Germany; University of Tennessee

**Keywords:** multiplex diagnostic assay, next-generation sequencing, pathogen genomics, targeted enrichment

## Abstract

Traditional diagnostic assays often lack sensitivity and can be difficult to multiplex across many pathogens. Next-generation sequencing (NGS) can overcome some of these problems but has limited application in the detection of low-copy-number pathogens in complex samples. Targeted genome capture (TGC) utilizes oligonucleotide probes to enrich specific nucleic acids in heterogeneous extracts and can therefore increase the proportion of NGS reads for low-abundance targets. While earlier studies have demonstrated the utility of this technology for detection of novel pathogens in human clinical samples, the capacity and practicality of TGC-NGS in a veterinary diagnostic setting have not yet been evaluated. Here we report the use of TGC-NGS assays for the detection and characterization of diverse feline pathogen taxa. We detected 31 pathogens comprising nine pathogen taxa in 28 felid samples analyzed. This included 20 pathogens detected via traditional PCR and 11 additional pathogens that had not been previously detected in the same samples. Most of the pathogens detected were sequenced at sufficient breadth and depth to confidently classify them at the species or subspecies level. Target nucleic acids were enriched from a low of 58-fold to 56 million-fold relative to host nucleic acids. Despite the promising performance of these assays, a number of pathogens detected by conventional PCR or serology were not isolated by TGC-NGS, suggesting that further validation is required before this technology can be used in lieu of quality-controlled standard assays. We conclude that TGC-NGS offers great potential as a broad multiplex pathogen characterization assay in veterinary diagnostic and research settings.

## INTRODUCTION

Despite considerable advancements in the characterization of genetic sequence data afforded by next-generation sequencing (NGS) technologies, significant limitations in applying them to the veterinary diagnostic setting remain. NGS can fail to detect low-copy-number pathogens in complex biological samples due to low pathogen/host nucleic acid ratios. For example, if 10% of mammalian cells (approximately 3 × 10^9^ bp per cell) in a blood sample are infected with a pathogen with a 10-kb genome, only 0.000033% of sequences in an NGS output file will be of pathogen origin. Additionally, pathogens isolated from clinical samples often have poor or only partially characterized genomes, can exist as genetically diverse populations within a single individual, or may be present in low abundance due to latency or stage of infection. This combination of small genome size, low relative copy number, and heterogeneous genomic content complicates pathogen detection from complex samples in the context of NGS diagnostic capabilities. For these reasons, virus isolation or culture methods for bacteria and fungi are often required prior to NGS to increase the proportion of on-target reads from clinical samples. These limitations prevent the adoption of NGS as a standard workflow in veterinary diagnostic laboratories.

Targeted genome capture (TGC) is a methodology that enriches specific genetic sequences within a heterogeneous mixture of DNA or RNA (1). Whole DNA extracts (or cDNA converted from RNA) is mixed with predesigned oligonucleotide probes under conditions that maximize hybridization to genetically similar sequences in the extract. Nontarget DNA does not bind to the probes and is subsequently removed, whereas DNA bound to the probes is eluted to achieve a higher concentration of target nucleic acids than was present in the original sample. Targeted genome capture has been used to enrich nucleic acids of interest from complex biological samples. Examples include the concentration of Wolbachia DNA (an intracellular bacteria) from whole-insect DNA extracts (2) and the enrichment of Yersinia pestis DNA from Black Death victims (3). Additionally, TGC has been recently used by Wylie et al. to demonstrate potential uses in detecting multiple viral taxa from human clinical cases, which represents an important step in adapting TGC technology for diagnostics or pathogen discovery in human samples (4).

Here we present the use of TGC-NGS to simultaneously detect and sequence multiple pathogens from domestic cats, bobcats, and mountain lions. We screened diverse sample types, including tissue culture fluids, blood, plasma, and swabs previously characterized for multiple pathogens using standard PCR or serological assays. Because some pathogens are common across feline species while others are species specific, we were able to evaluate the performance of these assays across several pathogen-host relationships. We designed two custom probe libraries to evaluate the ability of TGC-NGS to detect DNA and RNA from 10 viral and 1 bacterial taxa. The samples analyzed were derived from *in vitro* and *in vivo* sources covering a range of pathogen-to-host genomic ratios, from very low to very high. A bioinformatics pipeline was developed to identify host and pathogen sequences from the NGS output. Assay parameters were varied to assess the impact of probe concentration on enrichment efficiency, and unenriched libraries were analyzed by NGS to qualitatively assess the sensitivity of the NGS pipeline in detecting the presence of pathogens without enrichment.

Our report provides advances in investigating several important aspects of TGC assay design, including (i) the range of fold enrichment possible for targeted nucleic acids, (ii) comparison of TGC pathogen detection with traditional methods currently used by diagnostic laboratories, (iii) documentation that a single assay design can be applied to more than one host species, (iv) the applicability of TGC to characterize pathogens in the context of veterinary medicine, and (v) the ability of TGC-NGS to characterize intrahost genetic diversity of viral pathogens (4–6).

We detected 31 pathogens in 28 samples representing 9 of 11 targeted taxa, including 11 pathogens that had not been previously detected by traditional methods. The enrichment of target nucleic acids achieved by TGC ranged from 58-fold to a remarkable 56 million-fold. Subsequent analysis of tissues from cats with feline immunodeficiency virus (FIV) infection documented 5 logs of enrichment that occurred at the TGC processing step. Both DNA and RNA pathogens were detected in this study, although this method was more readily applied to the detection of DNA genomes. We observed poor performance of the assay under the following conditions: (i) samples with low pathogen loads, (ii) poorly preserved samples, or (iii) when pathogens lacked significant homology to probe sequences. We conclude that this approach to detecting and characterizing diverse pathogens from biological samples is a powerful and effective methodology with practical applications in veterinary clinical microbiology. Further side-by-side evaluations with clinical samples will be necessary to fully develop this method for high-throughput diagnostic use.

## RESULTS

### Detection and characterization of pathogens. (i) DNA pathogen detection.

Ten (of 16) DNA samples were known to have one or more target pathogens based on previous diagnostic assays (16 total pathogens) (Table 1, asterisks). At least one previously identified pathogen was detected in nine of these samples. All target pathogen taxa were detected except puma lentivirus clade A (PLVA), which was not sequenced in any of the three samples in which it was detected via nested PCR (estimated to represent less than 100 viral copies per one million cells, or 3.3 × 10^−9^ pathogen nucleotides/host nucleotide). Nine pathogens were detected that had not been detected prior to this study despite analysis by standard laboratory methods. The presence of all newly detected pathogens was reconfirmed by additional PCR analysis of the original samples. The breadth of coverage (percentage of each targeted genome region that was covered by sequencing reads) varied from 0 to 100%, with an average breadth of 84% among the 22 pathogens detected (Table 1). No reads could be confidently classified as belonging to novel feline pathogens.

**TABLE 1:** Detection of 22 pathogens and production of many genomic or subgenomic sequences by the TCG-NGS DNA assay^*a*^

Sample	Source of DNA	% Alignment of sequencing reads to indicated target pathogen^*b*^									
		FFV	FHV-1	FIVA	FIVC	PLVB	PLVA	FeLV^*c*^	M. haemo^*d*^	HTLV-like	Gamma
1	Whole blood	97 (5.9)*					ND*		23		
2	Whole blood	99 (13.6)				56*	ND*				
4	Whole blood	71*				72*			86*		
5	Cell culture			100 (672.3)*							
6	Cell culture				100 (3,872.6)*						
7	Whole blood				99 (16.4)*						
8	Cell culture		99 (2,212.0)*	74*		93 (9.5)*					
9	Cell culture		99 (2,170.5)*								
10	Whole blood		97 (18.2)						100 (724.2)		
11	Whole blood										
12	Whole blood	60	97 (7.3)								
13	Lymphoma	54		98 (72.0)							
14	Spleen					92 (5.3)		100 (15.9)*			
15^*e*^	PBMC										
16^*e*^	Cell culture

a Abbreviations: FFV, feline foamy virus; FHV-1, feline herpesvirus 1; FIVA, feline immunodeficiency virus clade A; FIVC, feline immunodeficiency virus clade C; PLVB, puma lentivirus clade B; PLVA, puma lentivirus clade A; FeLV, feline leukemia virus; M. haemo, Mycoplasma haemofelis; HTLV, human T-lymphotropic virus; Gamma, gamma herpesvirus; PBMC, peripheral blood mononuclear cells.

b Asterisks indicate results for samples with pathogens known to be present prior to this study. Values are the breadth (percentage) of each targeted genome region that was sequenced, indicated for all detected pathogens (ND, not detected). The mean read depth for samples with >90% sequencing breadth is indicated in parentheses. Dark shading indicates targets for pathogen discovery predicted to be present in some samples based on previous serological assays or clinical presentations.

c Only the long terminal repeat region of the genome was targeted.

d Only the 16S rRNA region of the genome was targeted.

e Negative control for all indicated pathogens.

### (ii) RNA pathogen detection.

Eleven of the 12 RNA samples sequenced had at least one target pathogen based on previous diagnostic assays (Table 2). Nine samples (including one negative control) were enriched according to the standard SureSelect protocol using the custom RNA probe library. One sample was enriched using half the recommended concentration of probes, and two were sequenced without enrichment (see Materials and Methods). Seven of 11 pathogens previously detected were sequenced, including four of four samples with previous detection of feline immunodeficiency virus (FIV) and three of three samples containing feline coronavirus (FeCoV), including the sample (sample 21a) subjected to half the concentration of TGC probes as other samples. Feline foamy virus (FFV) and feline leukemia virus (FeLV) were partially sequenced in a sample in which these pathogens had not been previously detected. Four pathogens that had been detected by serologic methods were not detected by TGC. This included three feline calicivirus (FCV) isolates and one feline herpesvirus 1 (FHV-1) isolate. The FCV and FHV-1 isolates were originally diagnosed via quantitative PCR (qPCR) using oronasal swab samples.

**TABLE 2:** Characterization of nine pathogens with >50% sequence breadth for most detected pathogens by the TGC-NGS RNA assay^*a*^

Sample	Source of RNA	% Alignment of sequencing reads to indicated target pathogen^*b*^							
		FFV	FHV-1^*c*^	FIVA^*d*^	FIVB	FIVC	FeCoV	FCV^*c*^	FeLV^*e*^
17	Cell culture					100*^f^			
18	Whole blood (enriched)					100*^f^			
18a	Whole blood (unenriched)					ND*			
19	Whole blood			82*					
20	Serum						34*		
21	Ascites (enriched)						62*		
21a	Ascites (enriched 1/2 baits)						54*		
21b	Ascites (unenriched)						ND*		
22	Serum		ND*		20*			ND*	
23	Plasma								
24	Oronasal swab	27*						ND*	100^*f*^
25^*g*^	Cell culture				

a Abbreviations: FFV, feline foamy virus; FHV-1, feline herpesvirus-1; FIVA, feline immunodeficiency virus clade A; FIVB. feline immunodeficiency virus clade B; FIVC, feline immunodeficiency virus clade C; FeCoV, feline coronavirus; FCV, feline calicivirus; FeLV, feline leukemia virus.

b Asterisks indicate results for samples with pathogens known to be present prior to this study. Values are the percentage of each targeted genome region that was sequenced, indicated for all detected pathogens (ND, not detected).

c Original detection made with oronasal swabs.

d Only the *gag* and *env* genes were targeted.

e Only the long terminal repeat region of the genome was targeted.

f Mean sequencing depth was greater than 10,000 reads per base.

g Negative control for all indicated pathogens.

Of the pathogens detected by the RNA assay, the breadth of coverage ranged from 20% to 100%, with a mean of 58.3% (Table 2). The depth of sequencing also varied greatly from a low of 30 reads/base for FFV (sample 24) to a high of 295,367 reads/base for FIV (sample 17). Neither of the pathogens in the two unenriched samples (FIV in sample 18a and FeCoV in sample 21b) were detected by NGS, although both of these pathogens were characterized in their paired, enriched RNA samples.

### DNA enrichment and sequencing.

**FIG 1:**
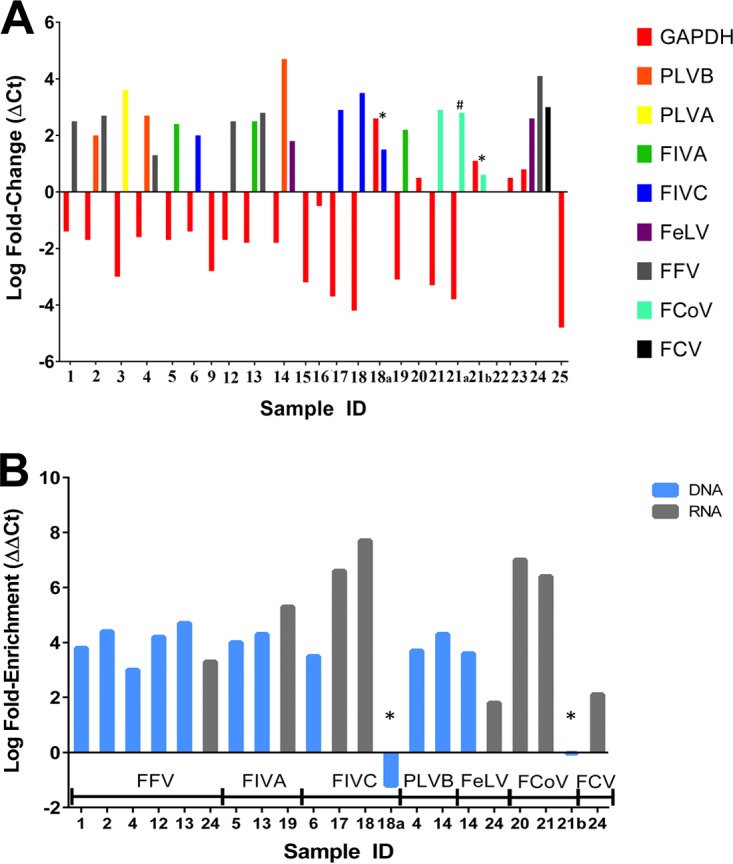
(A) Targeted genome capture results in the enrichment and depletion of pathogen and host nucleic acids, respectively. RT-PCR was performed pre- and postcapture for feline genomic DNA/cDNA (GAPDH) and individual pathogens pre- and postcapture. Two unenriched samples (*) demonstrated an increase of ≥1-fold in the relative abundance of the host GAPDH gene, compared to all of the enriched samples tested, in which GAPDH either decreased or remained approximately the same. One sample (#) was enriched with only half of the recommended capture probes but still demonstrated a depletion of host nucleic acids and an increase in pathogen nucleic acids. All pathogen nucleic acids measured increased in relative abundance postenrichment. Pathogen and host nucleic acids were measured only in samples with sufficient volume remaining before and after sequencing to conduct RT-PCRs. (B) Pathogen nucleic acids were enriched from 58-fold to 56 million-fold relative to host nucleic acids. The relative abundance of pathogen DNA did not increase in the two unenriched samples (*), but the corresponding paired, enriched pathogens increased over 1 million-fold. Samples in this figure are grouped by pathogen type: FFV, feline foamy virus; FIVA, feline immunodeficiency virus clade A; FIVC, feline immunodeficiency virus clade C; PLVB, puma lentivirus clade B; FeLV, feline leukemia virus; FCoV, feline corona virus; FCV, feline calicivirus.

All 12 DNA samples in experiment 1 demonstrated a relative decrease of host GAPDH (glyceraldehyde-3-phosphate dehydrogenase) gene copies (Fig. 1A, samples 1 through 16). Depletion of host DNA averaged 74-fold in DNA libraries relative to original DNA samples (range, 3.2-fold to 1,600-fold decrease). Conversely, all 13 pathogens evaluated pre- and post-TGC by real-time PCR (RT-PCR) demonstrated relative increases in copy number, averaging 386-fold postcapture enrichment (range, 20-fold to 50,000-fold increase). The overall enrichment of pathogen DNA relative to host DNA averaged 14,000-fold (range, 970-fold to 2.8 million-fold relative increase in abundance) (Fig. 1B).

The number of NGS reads per sample ranged from 0.64 to 3.1 million (median, 0.79 million) (Table 1). Two samples (samples 8 and 9) accounted for 35% of the total NGS reads generated for the 16 pooled DNA samples. These samples contained DNA extracted from high-titer *in vitro* cultures of feline herpesvirus and had the lowest percentage of reads map to the cat genome (3% and 1%, respectively). For all other DNA samples, the majority of NGS reads (51% to 82%) mapped to the cat genome. Each sample contained sequences that did not map to either the cat genome or the target pathogen reference sequences representing 20 to 46% of the total reads. We postulate that most of the unmapped reads correspond to feline genomic sequences that did not map to the cat genome reference sequence, which remains incompletely annotated.

### RNA enrichment and sequencing.

GAPDH RNA was decreased in postcapture libraries relative to that in original samples in six of the nine enriched RNA samples evaluated (Fig. 1A, samples 17 through 25). The average depletion of host RNA (cDNA) was 128-fold (range, 6.3-fold increase to 63,000-fold decrease). Three enriched samples had similar or slightly increased levels of GAPDH RNA postcapture. The average difference in postcapture target pathogen cDNA relative to the original samples was 1,000-fold for enriched samples (range, 158-fold to 12,500-fold increase). The overall abundance of pathogen cDNA relative to host cDNA increased by an average of 107,000-fold in postcapture samples (range, 58-fold to 56 million-fold) (Fig. 1B).

Interestingly, FeCoV in sample 21a, which was enriched using only half of the recommended concentration of baits, had values for the depletion of host cDNA and enrichment of pathogen cDNA similar to those of sample 21, which used the full concentration of baits during the hybridization (capture) step in the protocol (Fig. 1A). The two unenriched samples (18a and 21b) were the only samples evaluated by RT-PCR that did not have a relative increase in abundance of pathogen cDNA relative to the host (Fig. 1B).

The number of reads per RNA sample ranged from 2.7 million to 85.1 million (median, 24.5 million) (Table 3). Of these, the proportion of reads for each sample that mapped to the cat genome ranged from 0.7% to 65.3% (median, 41.8%). The proportion of reads that mapped to the pathogen reference sequences ranged from less than 0.1% to 96.3% (median, 1.0%). The proportion of reads that did not map to the cat genome or the target pathogen sequences accounted for 3% to 98.9% of the sample reads (median, 43.6%).

### FIV enrichment quantification in experimental samples.

**FIG 2:**
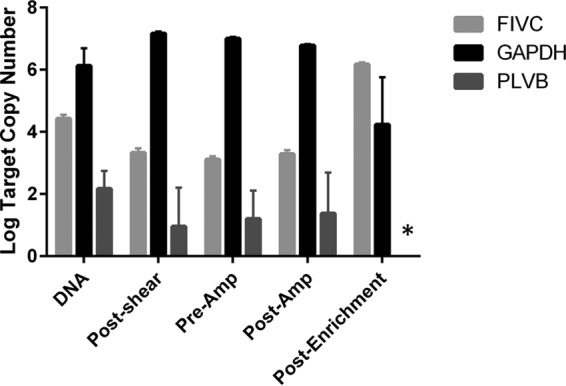
Enrichment drastically increases the ratio of target pathogen nucleic acids to host nucleic acids in experimental samples. Quantitative RT-PCR results demonstrate an increase in the abundance of targeted nucleic acids (FIVC) and a decrease in nontargeted nucleic acids (PLVB and GAPDH) during the enrichment step in the protocol. *, PLVB was undetectable in postenrichment samples. Amp, amplification.

The log copy numbers of FIV and GAPDH copies remained relatively unchanged throughout the course of the preenrichment library preparation steps, with FIV copies present at a level approximately 3-log fold lower than that of GAPDH copies (mean FIV copy number = 3.54, SD = 0.60; mean GAPDH copy number = 6.77, SD = 0.46) (Fig. 2). However, the enrichment and subsequent PCR resulted in an increase of FIV copies and a decrease of GAPDH copies in each sample. The average enrichment of FIV relative to GAPDH was 147,000-fold. PLVB was originally detected at a very low copy number (100 copies per million cells) by qPCR but was not detected in any of the final enriched samples. FIV probes included in a second set of DNA probes (DNAv2) included sequences that shared 63% pairwise identity to PLV proviral genomic regions present in extracted tissues (alignment available upon request).

## DISCUSSION

Next-generation sequencing is transforming many fields of investigation, including diagnostic evaluations and novel pathogen discovery. The current limitations of NGS application for these purposes include (i) sensitivity of detection of minute pathogen genome nucleotide ratios within complex eukaryotic host samples, (ii) bioinformatics pipelines that can efficiently detect NGS output data for veterinary pathogens, (iii) expertise required to design and conduct pipelines for biological problems, and (iv) expense for high-throughput analysis.

While targeted enrichment is becoming widely used to screen for polymorphisms within human genomic loci associated with cancers and inherited disorders (7–9), its uses for multiplex pathogen characterization are still not widespread and, to our knowledge, have never been applied to veterinary pathogens. Wylie et al. (10) recently reported the development of a comprehensive probe library for pathogen diagnostics and discovery that holds tremendous potential. Here, we have adapted a similar strategy for veterinary clinical and discovery purposes. We have demonstrated the potential for successfully implementing the TGC-NGS approach and have also identified current limitations to widescale application.

This is the first application of targeted genome capture and next-generation sequencing as a large multiplex pathogen screening tool for animal pathogens. The custom capture probe libraries we designed include divergent DNA and RNA sequences selected to represent the known diversity of each target pathogen (Table 3; see Table S1 in the supplemental material). In total, 31 pathogens were detected in 28 samples, including 20 previously characterized pathogens and 11 pathogens that were previously undetected in these samples, despite being subjected to standard diagnostic analyses (Tables 1 and 2). The enrichment of pathogen nucleic acids relative to that of the host was as high as 56 million-fold over the starting concentrations. This level of enrichment enabled greater than 50% sequencing breadth for the genomic regions targeted by probes for 27 pathogens, from 13 viral or bacterial species or subspecies that were partially or completely sequenced.

**TABLE 3:** Pathogens specifically targeted for enrichment and sequencing in the TGC-NGS assays

Pathogen family	Infectious agent	Genome	Rationale for inclusion
Caliciviridae	Feline calicivirus^*a*^	7.7 kb, RNA	Persistent infection; rapidly mutates; pathogenicity varies greatly by strain
Coronaviridae	Feline coronavirus^*a*^	29.1 kb, RNA	Suspected genetic basis for switch from apathogenic to pathogenic phenotype
Herpesviridae	Feline herpesvirus^*a*^^,^^*b*^	134 kb, DNA	Potential cofactor in lymphoma; highly prevalent; latent infection; improved detection methods needed
Mycoplasmataceae	Mycoplasma haemofelis^*a*^^,^^*b*^	1.1 Mb, DNA^*c*^	Chronic transmissible infections possible; high morbidity and mortality if untreated; broad host range
Retroviridae^*d*^	Feline leukemia virus^*a*^^,^^*b*^	9.6 kb, RNA^*c*^	Endogenous and exogenous forms; latent yet transmissible
	Feline immunodeficiency virus^*a*^^,^^*b*^	9.1 kb, RNA	Latent infection; mutates rapidly; role in a variety of disease states
	Puma lentivirus^*a*^^,^^*b*^	9.1 kb, RNA	Detection assays lack sensitivity; cross-species transmission and host range expansion documented
	Feline foamy virus^*a*^^,^^*b*^	11 kb, RNA	Suspected cofactor in feline diseases; broad host range

a Pathogen taxon included in the RNA assay.

b Pathogen taxon included in the DNA assay.

c Only a portion of the genome was targeted.

d The DNA copy of the RNA genome (provirus) was targeted in the DNA assay. Table S1 contains the full list of pathogen taxa included in the custom probe libraries.

A specific analysis of enrichment from lymph nodes of cats exposed to FIV clade C (FIVC) and PLVB documented enrichment of the targeted pathogen, FIVC, but no enrichment of the related but distinct virus, PLVB, which was not targeted in the DNA v2 probe set (Fig. 2). Analysis of enrichment at each step of the analytical process documented that, as expected, FIVC and host DNA were detected at each step of the protocol, but enrichment occurred following TGC. In contrast, PLVB was detectable in all four samples preenrichment but was not detectable in any of the samples postenrichment, consistent with the lack of PLVB probes in the DNAv2 probe library. The results suggest that the homology between probes and targeted nucleic acids needs to be greater than the 63% pairwise identity shared between PLVB and FIVC in the region screened by the RT-PCR primers used to quantify enrichment.

Current diagnostic assays that can simultaneously detect and genetically characterize pathogens are rarely multiplexed beyond a few targets in one assay. In comparison, the TGC-NGS assays implemented here produced sequence data for 9 of 11 target pathogen taxa known to be present in at least one sample (seven in the DNA assay and six in the RNA assay with four taxa sequenced by both [Tables 1 and 2]). Future TGC designs will include probes targeting a much broader range of pathogen taxa (for example, see reference 4). The assay was also successful at detecting and sequencing pathogens from diverse sample sources, including single and pooled nucleic acid extracts, with up to three pathogens per sample originating from experimental (*in vitro* and *in vivo* infections) and clinical (natural infections) samples from different host species. We additionally documented that variations in probe concentration can be optimized to decrease the per sample cost of targeted enrichment. Further, our analysis demonstrates that one probe library can be used to detect pathogens in three different species, providing proof of concept of streamlined diagnostic reagents/protocols for a range of species. With further optimization, and with increasingly more affordable NGS technologies, we feel that this approach can expand the capacity to simultaneously sequence a broad range of pathogens in clinical and research settings and potentially revolutionize veterinary diagnostic analysis.

Most diagnostic tests based on nucleic acid detection (as opposed to those that detect antigens or antibodies) amplify short, conserved regions of pathogen genomes. The output of these assays is either “presence versus absence” data (i.e., real-time PCR) or short sequences that provide little to no information about the diversity of the isolates present. While the percentage of on-target reads was generally low, the breadth and depth of sequencing for target pathogens in most samples in our analysis were much greater than that produced through conventional PCR-based assays. For each of the pathogens detected by our assay, enough of the genome was sequenced to confidently classify pathogens at the species or subspecies level, which is especially valuable for potentially pathogenic organisms that are not part of routine multiplex diagnostic screens (contigs/alignments available upon request). In several cases, full-genome sequences were produced and sequencing was deep enough to see evidence of intrahost genetic diversity (for example, see Fig. S1, which demonstrates intrahost diversity in sample 13). TGC-NGS assays could therefore not only function as powerful multiplex diagnostic tools but also produce sequencing output suitable for use in studies on epidemiology, drug resistance, phylogenetics, evolution, and many other fields that utilize molecular data from pathogens.

By estimating the relative abundance of host and pathogen nucleic acids pre- and postenrichment, we demonstrated a substantial increase in the pathogen-to-host genomic content of postenrichment samples (Fig. 1B). Consistent with our expectations, all samples for which enrichment was estimated showed an increase in the relative abundance of target pathogen nucleic acids except for the two unenriched samples. Our RT-PCR data demonstrate that the enrichment was the result of both depletion of host genetic material (negative fold change in GAPDH) and an increase in target pathogen nucleic acids (positive fold change in pathogen DNA/RNA) (Fig. 1A). Mean enrichment values of 250,000-fold and 9,100,000-fold with the DNA and RNA probes, respectively, and 147,000-fold for FIV contained in tissues clearly demonstrate the ability of the targeted capture probes to dramatically alter the relative abundance of specific nucleic acids across a range of sample types and diverse pathogen taxa.

At least one pathogen was detected in 15 of 18 enriched clinical samples. This indicates that the majority of targeted pathogen taxa are detectable at physiologic levels of infection. The two paired, enriched versus unenriched samples analyzed in the RNA assay provide a qualitative estimate of the impact of enrichment on detection and sequencing outcomes. The full genome of FIVC was sequenced at an average depth of 13,982 reads/base in sample 18 (enriched), while the number of FIVC reads in its paired sample, 18a (unenriched), was below the threshold for detection. Similarly, 66% of the FCoV genome was sequenced at an average depth of 5,212 reads/base in sample 21 (enriched), but the number of reads mapped to FCoV in sample 21b (unenriched) was again below the threshold for detection. By comparison, FCoV was detected in sample 21a, which was enriched using half of the recommended probe concentration (see Materials and Methods), although the coverage and depth of sequencing was less than in sample 21. From this we conclude that enriching nucleic acids with TGC prior to NGS can increase the ability to detect and sequence pathogens in heterogeneous samples.

While the results reported here suggest that the TGC-NGS methodology holds great promise as a broad pathogen detection assay, our study also reveals some limitations. Ideally, all individual DNA/cDNA libraries pooled for sequencing would produce nearly equal numbers of NGS reads, and a high proportion of the total reads for each sample would correspond to pathogen sequences. However, in this study, the total number of reads varied widely and the percentage of on-target reads was low for most *in vivo* samples (Tables 4 and 5). This is likely the result of multiple factors, including differences in sample quality, differences in pathogen copy number among samples, and an unequal number of probes targeting different pathogen taxa in the custom probe libraries. Despite this limitation, it is encouraging that although the percentage of on-target reads for most samples was low, the number of pathogen reads was sufficient to accurately characterize the pathogens in most samples analyzed.

**TABLE 4:** Reads per sample that mapped to the cat genome or pathogen reference sequences or that were unmapped*^a^* (corresponding to results shown in Table 1)

Sample	Species	Total no. of reads	No. (%) of cat genomic reads	No. (%) of target pathogen reads	No. (%) of unmapped reads
1	Puma	719,175	480,100 (66.8)	3,197 (0.4)	235,878 (32.8)
2	Puma	874,893	572,833 (65.5)	5,003 (0.6)	297,057 (34.0)
3	Puma	628,990	403,370 (64.1)	1,692 (0.3)	223,928 (35.6)
4	Puma	763,056	509,932 (66.8)	4,630 (0.6)	248,494 (32.6)
5	Domestic cat	717,024	529,156 (73.8)	55,288 (7.7)	132,580 (18.5)
6	Domestic cat	815,416	411,768 (50.5)	239,758 (29.4)	163,890 (20.1)
7	Domestic cat	716,472	569,956 (79.6)	5,364 (0.7)	141,152 (19.7)
8	Domestic cat	2,716,103	79,269 (2.9)	2,179,068 (80.2)	457,766 (16.9)
9	Domestic cat	3,099,809	24,954 (0.8)	1,890,404 (61.0)	1,184,451 (38.2)
10	Bobcat	912,974	600,982 (65.8)	45,223 (5.0)	266,769 (29.2)
11	Bobcat	645,070	427,663 (66.3)	10,892 (1.7)	206,515 (32.0)
12	Domestic cat	797,028	654,712 (82.1)	9,343 (1.2)	132,973 (16.7)
13	Domestic cat	888,858	593,644 (66.8)	10,523 (1.2)	284,691 (32.0)
14	Puma	752,426	396,665 (52.7)	6,277 (0.8)	349,484 (46.4)
15	Domestic cat	808,993	636,270 (78.6)	2,461 (0.3)	170,262 (21.0)
16	Domestic cat	852,370	679,878 (79.8)	2,974 (0.3)	169,518 (19.9)

**TABLE 5:** Reads per sample that mapped to the cat genome or pathogen reference sequences or that were unmapped*^a^* (corresponding to results shown in Table 2)

Sample	Species	Total no. of reads	No. (%) of cat genomic reads	No. (%) of target pathogen reads	No. (%) of unmapped reads
17	Domestic cat	28,806,008	210,135 (0.7)	27,737,588 (96.3)	858,285 (3.0)
18	Domestic cat	20,017,045	3,219,403 (16.1)	1,315,088 (6.6)	1,904,315 (77.3)
18a	Domestic cat	85,104,702	16,352,937 (19.2)	43,823 (0.1)	68,707,942 (80.7)
19	Domestic cat	2,700,579	548,220 (20.3)	80,482 (3.0)	2,071,877 (76.7)
20	Domestic cat	15,832,275	10,238,858 (64.7)	40,930 (0.3)	5,552,487 (35.1)
21	Domestic cat	69,703,797	38,143,416 (54.7)	3,831,026 (5.5)	27,729,355 (39.8)
21a	Domestic cat	16,660,006	9,987,838 (60.0)	278,701 (1.7)	6,393,467 (38.4)
21b	Domestic cat	69,376,796	38,770,753 (55.9)	30,430 (0.0)	30,575,613 (44.1)
22	Domestic cat	20,259,245	5,861,761 (28.9)	47,278 (0.2)	14,350,206 (70.8)
23	Domestic cat	32,424,304	21,171,586 (65.3)	10,222 (0.0)	11,242,496 (34.7)
24	Domestic cat	19,678,144	10,801,415 (54.9)	382,448 (1.9)	8,494,281 (43.2)
25	Domestic cat	29,313,305	299,572 (1.0)	9,944 (0.0)	29,003,789 (98.9)

Some pathogen taxa were consistently detectable and achieved moderate to good sequencing breadth/depth in multiple samples. For example, FIV was detected in nine samples with an average sequencing coverage of 86%. In contrast, PLVA and FCV were not detected in any of the samples in which they were known to be present (Tables 1 and 2). This is despite the fact that PLVA was enriched 4 million-fold in sample 3 and that FCV was enriched 135-fold in sample 24 (Fig. 1A and B). PLVA and FCV were undetectable by RT-PCR both pre- and postenrichment in the other samples known to contain these pathogens. During analysis of experimental tissues, PLVB was not enriched with probes that had low pairwise identity to viral sequences (Fig. 2). These results suggest that some very low-copy-number pathogens are not consistently detectable by these assays as they were implemented here, and probe homology to pathogen genomes can significantly impact detection. Future uses of the assay could utilize deeper sequencing, degenerate probe sets, and/or an optimized enrichment protocol to improve the sensitivity of the assay for very low-copy-number pathogens.

While TGC-NGS proved useful for sequencing large segments of pathogen genomes, which may be a benefit for certain applications, the enrichment range was not linear, and therefore this technology is not currently useful for replacing quantitative methods such as qPCR or viral titration. It is feasible that with further optimization and development of quality control procedures, it will be possible to develop more quantitative analyses. Similarly, a robust prospective comparison of TGC-NGS sensitivity compared to traditional diagnostic assays across different sample sources and qualities could enable the development of standardized power calculations such as those routinely applied to microbiome studies (11).

The RNA TGC assay failed to detect four pathogens from the enriched samples (Table 2). This was despite the use of deeper sequencing techniques in the RNA assay (12 samples multiplexed on a HiSeq instrument) than in the DNA assay (16 samples multiplexed on a MiSeq instrument). We believe this is likely due to low-quality RNA isolated from samples more than 3 years old that were not stored in RNA storage buffer. Our results would indicate that in addition to the starting pathogen genome concentration, sample quality is an important predictor of TGC-NGS success.

The cost for development of probes, NGS, labor required to conduct the study, and other reagents is estimated to be approximately $450 to $550 per sample at the time that this procedure was conducted. Going forward, the costs for this technology are likely to rapidly decrease to below $200/sample. Current diagnostic assays using traditional methods typically run $50 to $250/test, depending upon the type of analysis required. Since TGC-NGS is likely to be able to identify a wide spectrum of pathogens with a single analysis, optimized development of this technology is likely to be within an affordable range in the near future.

A secondary objective of this study was to explore the TGC-NGS assay for potential use in discovering novel pathogens. Previous serological results suggested that bobcats in Colorado may be infected with PLV-like and FFV-like viruses known to circulate in other bobcat populations or in sympatric felids (A. Bleiholder and M. Lochelt, unpublished data). Previous attempts to identify these viruses have been unsuccessful, and therefore two samples were included to determine if the TGC-NGS assay could detect divergent or low-copy-number pathogens undetectable by conventional PCR. Although we included diverse PLV and FFV sequences in the capture probe library, we did not detect previously undescribed pathogens using this approach. Two other samples, each containing pooled DNA from two domestic cats with either lymphoma or T-cell leukemia, were also included to search for infectious etiologies for these common feline disease states. Viral pathogens produce clinically similar pathologies in other species, e.g., T-cell leukemia can result from human T-lymphotropic virus type 1 (HTLV-1) infection in humans and many species harbor gammaherpesviruses that initiate neoplastic conditions such as lymphomas. This analysis did not reveal pathogen genomes consistent with these hypothesized viral etiologies. It is likely that additional optimization of probes and technical aspects of the process will provide greater resolution of novel pathogens using this technology.

### Conclusions.

We have shown that TGC-NGS assays can function as broad multiplex pathogen detection assays for animal pathogens. We also demonstrated that hybridization to targeted capture probes can enrich pathogen nucleic acids up to 56 million-fold relative to host genes and that full genome sequences were retrieved from both clinical and experimental samples from three different host species. A significant number of pathogens were identified that were not readily detected using more traditional veterinary diagnostic technologies, and we documented that bioinformatics analysis alone was not sufficient to detect pathogens from unenriched samples. We identified the limitations of this technology related to very low starting pathogen concentrations and poor sample quality. Optimizing capture probes and implementing stringent quality control measures prior to NGS could enhance the utility of this technology for large-scale diagnostic use. We also recognize that while promising, NGS-based assays such as those described here require more investigation before they can be used in place of traditional diagnostic procedures, and therefore we recommend that laboratories adopting NGS-based assays do so alongside traditional methods until protocols are fully validated.

Importantly, our data illustrate that probe libraries could simultaneously screen for all known pathogens of a given host species (i.e., feline and canine pan-pathogen probe libraries) or group of host species (i.e., livestock and companion animal probe libraries). The TGC-NGS approach could also be modified for specific research or clinical applications. For example, the number of probes per target pathogen could be increased if the goal is to investigate genetic diversity or rare genetic variants among a small set of pathogens. This may improve the capture efficiency and sequencing depth for each pathogen, enabling analyses into, for example, molecular evolution and intrahost diversity. Alternatively, by targeting only conserved regions of pathogens, instead of full-length genomes, more target pathogens could be included in a single capture probe library, as suggested by others (4). This could allow investigations that “cast a big net” when screening for pathogens within individuals or populations and allow for the discovery of unidentified pathogens of importance to the veterinary community.

## MATERIALS AND METHODS

### Design of capture probes.

Probes were designed for the capture of nucleic acids from the viral and bacterial feline pathogens listed in Table 3 and in Table S1 in the supplemental material. Target pathogens were chosen because they fit one or more of the following characteristics: (i) the pathogen is highly relevant to feline health, yet current assays often fail to detect or completely characterize infection due to latency and/or high mutation rate; (ii) a relatively small genome size allowed redundancy in the capture design, providing enhanced sensitivity to diversity within the taxa; and (iii) the virus family is involved in analogous pathological processes (i.e., neoplasia or anemia) in other host species. The last attribute was included to allow potential discovery of pathogens that have not yet been associated with a particular disease syndrome.

Full-length and partial genetic sequences representing the known diversity of each target pathogen were downloaded from GenBank (www.ncbi.nlm.nih.gov/GenBank/). Nucleotide alignments were constructed using default parameters in Muscle (12). Maximum-likelihood (ML) trees were built in MEGA v.5, using the Hasegawa-Kishino-Yano model of nucleotide substitution (13) (trees available upon request). Rate variation among sites was estimated with a two-category gamma distribution, allowing for invariant sites. Cluster support was estimated with 100 bootstrap replicates. One or more sequences were selected arbitrarily for the probe sequence library from each polytomy and each monophyletic cluster of terminal nodes.

All sequences in the probe library were cross-referenced to the domestic cat (Felis catus) genome using the BLAST-like Alignment Tool (BLAT) (14) (the bobcat [Lynx rufus] and mountain lion [Puma concolor] genome sequences have not yet been reported). Sequences with greater than 80% homology to the cat genome (Felis_catus 6.2, GenBank assembly accession no. GCA_000181335.2) were removed from the probe library to prevent nonspecific capture of host chromosomal DNA. This step was especially important for the exclusion of probes complementary to the endogenous feline leukemia virus (FeLV) sequences that exist in the domestic cat genome.

We designed separate probe libraries for the enrichment of pathogen DNA and RNA (Table S1; see “Sample preparation” below). Probe libraries produced by Agilent Technologies, Inc. (Santa Clara, CA), contained approximately 55,000 RNA oligonucleotides, 120 bp in length, complementary to the DNA or RNA sequences of interest. For each probe library, the sequences selected from phylogenetic analyses described above were uploaded to eArrayXD capture probe design software (Agilent Technologies, Inc., Santa Clara, CA). Each set of probes was designed at 4× coverage of target sequences (individual 120-bp probes tiled every 30 bases). Although the two probe libraries were used separately for the identification of pathogen DNA or RNA, each library was designed with some probes complementary to both forms of nucleic acids for possible combined DNA/RNA enrichment.

After the initial screen of samples with the DNA probe library, a second set of DNA probes (DNAv2) was designed to improve capture efficiency by decreasing off-target capture. The DNAv2 probe library contained all of the domestic cat pathogens included in the first version but did not target pathogens specific to bobcats and mountain lions and had less redundancy. The sequences included in each probe library design are available upon request.

### Sample preparation.

Samples were selected to represent the following categories of pathogen status (Table 1): (i) samples known to contain one or more target pathogens based on previous diagnostic tests, (ii) samples presumed to be negative for pathogens contained in the corresponding DNA/RNA probe library for that experiment (i.e., specific-pathogen free cats, cell cultures), (iii) samples from animals with clinical presentation or serological results suggestive of pathogen involvement but for which no infectious etiology has been identified, and (iv) samples from cats experimentally exposed to feline immunodeficiency virus (FIV) and puma lentivirus (PLV). All samples were acquired from existing frozen sample archives. No live animals were used or sampled for this study.

### (i) DNA library preparation (16 samples).

DNA was extracted from blood, tissue, or cell culture samples from domestic cats, bobcats, and mountain lions using the DNeasy blood and tissue kit (Qiagen, Inc., Valencia, CA). For each sample, 3 μg of single or pooled DNA extracts was used to construct the DNA library according to the SureSelectXT automated target enrichment for Illumina paired-end multiplexed sequencing protocol (Agilent Technologies, Inc., Santa Clara, CA). Briefly, each DNA extract was sheared to produce short DNA fragments (∼150 to 200 bp). Standard Illumina adaptors were ligated to the ends of each fragment, and adaptor-specific primers were used to randomly amplify the DNA libraries prior to enrichment (4 to 6 PCR cycles).

### (ii) RNA library preparation (16 samples).

RNA was extracted from *in vivo* (blood, peritoneal ascites, oral swabs) or *in vitro* (viral culture supernatant) samples using a viral RNA kit (Qiagen, Inc., Valencia, CA). For each sample, a minimum of 1 μg RNA was used to construct the RNA library according to the SureSelect RNA enrichment for Illumina paired-end multiplexed sequencing protocol (Agilent Technologies, Inc., Santa Clara, CA). The RNA library preparation protocol was similar to that for DNA, except that after fragmentation, double-stranded cDNA (ds-cDNA) was produced from the positive-sense RNA present in the extract. The isolated ds-cDNA was then used as the template for adaptor ligation and amplification as described above. Four samples did not pass pre-NGS quality control analyses (i.e., due to low concentration or quality), and therefore only 12 of 16 original RNA samples were sequenced.

### (iii) DNA preparation for tracking enrichment (4 samples).

DNA was extracted from mesenteric lymph nodes from four domestic cats that had been experimentally coinfected with FIV and PLV for a viral pathogenesis study (15). For each sample, a DNA library was prepared and enriched with the DNAv2 probes as described above. These samples were used for quantifying host and pathogen nucleic acids at each step of the library preparation and enrichment protocols (see “Enrichment estimation” below).

### Target enrichment and sequencing.

DNA/cDNA libraries were hybridized to their respective custom capture probe libraries for 24 h at 65°C, and unbound DNA was removed by washing as recommended by the probe manufacturer (16). The enriched DNA/cDNA was then separated from the capture probes and isolated. Each DNA/cDNA library was barcoded via ligation of standard 6-mer oligonucleotides (Illumina, Inc., San Diego, CA) for multiplex sequencing. The concentration of each library was assessed using flourometric (Qubit 2.0; Thermo Fischer Scientific, Inc., Grand Island, NY) and electrophoretic (Bioanalyzer; Agilent Technologies, Inc., Santa Clara, CA) methods, and the libraries were pooled in equal concentrations for sequencing. The DNA libraries (*n* = 16) and cDNA libraries (*n* = 12) were pooled separately, and a nonindexed positive control was added to the samples at a 1% concentration (bacteriophage PhiX-174 DNA). DNA libraries during the first experiment were sequenced as a 16-sample multiplex with 150-bp paired-end reads on a MiSeq instrument (Illumina, Inc., San Diego, CA). The cDNA libraries were sequenced as a 12-sample multiplex with 100-bp paired-end reads on a HiSeq instrument (Illumina, Inc., San Diego, CA).

### Enrichment estimation.

We used real-time PCR (RT-PCR) to quantify the abundance of the feline GAPDH (glyceraldehyde 3-phosphate dehydrogenase) gene in pre- and postenrichment samples according to established protocols (17). GAPDH genomic DNA or cDNA was quantified using primers from reference 18 with the following 25-μl reaction mixture: 12.5 μl SsoFast EvaGreen SuperMix (Bio-Rad Laboratories, Inc., Hercules, CA), 1 μl GAPfwd primer (10 μM), 1 μl GAPrev primer (10 μM), 8.5 μl water, and 2 μl template (∼40 ng). The reaction conditions included an initial denaturation at 95°C for 3 min, followed by 40 cycles of 95°C for 10 s and 60°C for 10 s. This was followed by a melt curve in which the temperature was increased from 65°C to 95°C in 0.5°C increments using 10-s intervals.

The relative abundances of feline calicivirus (FCV), feline herpesvirus 1 (FHV-1), FIVA, and FIVC were measured with established RT-PCR protocols (10, 19, 20). PLVA proviral DNA was measured using the following RT-PCR mixture: 12.5 μl SsoFast EvaGreen SuperMix, 1 μl 8083F (10 μM) (5′-GCAGCCCTGACGGTATCC-3′), 1 μl 8165R (10 μM) (5′-GCAGTCTCCTCTGAACAATCC-3′), 5.5 μl water, and 5 μl (100 to 500 ng) template. The reaction conditions included an initial denaturation step at 95°C for 3 min, followed by 40 cycles of 95°C for 5 s and 62°C for 10 s. This was followed by a melt curve with the same parameters as described above for GAPDH. PLVB proviral DNA was measured using the same PCR mixture and conditions with 1 μl each of the following primers: 643F (10 μM) (5′-CTGTCTGTCATGGGGAATGAGT-3′) and 773R (10 μM) (5′-GTCCTGTAGCTACCAAGGCAA-3′).

Feline foamy virus viral (RNA) and proviral (DNA) loads were measured using the following RT-PCR protocol: 10 μl iTaq Universal Probes Supermix (Bio-Rad Laboratories, Inc., Hercules, CA), 1 μl FFVgag-F (10 μM) (5′-GGACGATCTCAACAAGGTCAACTAAA-3′), 1 μl FFVgag-R (10 μM) (5′-TCCACGAGGAGGTTGCGA-3′), 0.2 μl FFVgag-TM probe (10 μM) (5′-AGACCCCCTAGACAACAACAGCAACACT-3′), 5.8 μl water, and 2 μl template (40 to 100 ng). The reaction conditions included an initial denaturation at 95°C for 3 min, followed by 40 cycles of 95°C for 10 s and 60°C for 30 s.

Fold enrichment was calculated by comparing RT-PCR threshold cycle (*C_T_*) values for preenrichment (i.e., DNA extracts or cDNA) versus postenrichment (prepped libraries) samples via the ΔΔ*C_T_* method. Log_10_-transformed fold change (Δ*C_T_*) and fold enrichment (ΔΔ*C_T_*) values were graphed using GraphPad Prism v4 (GraphPad Software, San Diego, CA). The RT-PCRs and fold enrichment calculations were performed only on the subset of samples that had a sufficient sample volume remaining of pre- and postenrichment nucleic acids. Fold enrichment was similarly estimated at each step in the library preparation and enrichment protocol from FIV-containing samples enriched with DNAv2 baits.

In order to estimate the impact of enrichment on the sequencing output for targeted pathogens, 2 of the 12 cDNA libraries were sequenced without enrichment, and one was enriched using only half of the recommended concentration of capture probes. Each of these three libraries originated from the same RNA extraction as a normally enriched cDNA library that was also processed and sequenced in parallel (Tables 2 and 4).

### Bioinformatics analysis.

NGS reads were preprocessed by trimming known sequence contaminants from all sequences. Reads were then aligned to the Felis catus genome using BWA short-read alignment software (21). The reference sequence used for this experiment was assembly felCat5, which was obtained from the UCSC genome browser (https://genome.ucsc.edu). The genome was indexed by using the BWA-SW algorithm (22). Reads were considered successfully mapped if the read and its mate successfully aligned according to BWA. The mean and standard deviation of depth, coverage, total number of alignments, and total number of reads that did not successfully align were calculated for every chromosome of the assembly using an in-house script.

All reads that did not successfully align to the cat genome given the specified criteria were aligned to the nucleic acid sequences in the corresponding capture probe library. The successful alignment criterion was whether the read itself successfully aligned to any pathogen in the database according to BWA. The pathogen database was indexed using the BWA-backtrack algorithm. Like the alignment of reads to Felis catus, the same analytics were used to postprocess the alignments to each pathogen in the database. For alignments to both the Felis catus genome and the database of pathogens, all possible alignments according to BWA were considered, thus entailing that a read can have multiple alignments.

Some reads mapped to pathogen reference sequences even in the absence of pathogens being present in that sample. This was likely the result of spurious mapping of nonpathogen reads in BWA and contamination during DNA/cDNA library preparation, similar to results recently reported by Wylie et al. (4). To differentiate true pathogen reads from false-positive reads, we calculated a “positive threshold value” by using the number of reads that aligned to pathogen reference sequences in the negative-control samples. The positive threshold value was set at 100 times the proportion of total reads that mapped to each pathogen taxa in the negative-control samples. Thus, a positive threshold value was calculated for each pathogen taxa in each (DNA and RNA) experiment. A sample was considered positive for a given pathogen taxon if the proportion of reads that mapped to the pathogen reference sequences exceeded the positive threshold value for that taxon.

### Accession number(s).

The alignment files associated with this study can be found under BioProject PRJNA383388 in the SRA archive.

## Supplementary Material

Supplemental material
