# Supplementary material for: Targeted Enrichment for Pathogen Detection and Characterization in Three Felid Species
Source: J Clin Microbiol. 2017 May 23;55(6):1658–70. doi: 10.1128/JCM.01463-16 (PMC5442522; doi:10.1128/JCM.01463-16)
Supplement: Supplemental material [file supp_55_6_1658__index.html]

Targeted Enrichment for Pathogen Detection and Characterization in Three Felid Species — Supplemental material 

# Targeted Enrichment for Pathogen Detection and Characterization in Three Felid Species

## Supplemental material

- Supplemental file 1 -

  Table S1 (Number of probes targeting each pathogen in the custom probe libraries)

  PDF, 455K
- Supplemental file 2 -

  Fig. S1 (Reads mapped to FIVA demonstrate intrahost diversity within sample 13)

  PDF, 150K
