# Supplementary material for: Targeted Enrichment for Pathogen Detection and Characterization in Three Felid Species
Source: J Clin Microbiol. 2017 May 23;55(6):1658–70. doi: 10.1128/JCM.01463-16 (PMC5442522; doi:10.1128/JCM.01463-16)
Supplement: Supplemental material [file JCM.01463-16_zjm999095505s1.pdf]

Pathogen Taxa	# Probes DNA	# Probes RNA
Annaplasma phagocytophilum		1296[1]
Bovine immunodeficiency virus	283[2]	
Bovine leukemia virus		787[1]
Chicken anemia virus		1282[2]
Equine infectious anemia virus	275[2]	
**Feline calicivirus**	2594[2]	6192[2]
**Feline coronavirus**	10161[2]	14033[2]
**Feline foamy virus**	3566[2]	6989[2]
Feline gamma herpesvirus		1054[1]
**Feline herpesvirus**	9054[2]	574[1]
**Feline immunodeficiency virus A, B, C**	3888[2]	5202[2]
**Feline leukemia virus**	143[1]	227[1]
Feline morbilivirus		148[1]
Gallid herpesvirus-2		994[1]
Gamma herpesviruses	1430[1]	
Human herpesvirus-6		623[1]
Human T-lymphotropic virus	1164[2]	3207[2]
Infectious salmon anemia virus		1148[1]
Lion lentivirus	1155[2]	
Murine leukemia virus		2846[2]
Mycloplasma haemofelis	274[1]	796[1]
Mycoplasma haememinutum	227[1]	970[1]
Mycoplasma turicensis	219[1]	886[1]
**Puma lentivirus A & B**	17731[2]	
Ricketsia felis		257[1]
Simian immunodeficiency virus	639[2]	
Simian T-lymphotropic virus	1802[2]	3623[2]

Table S1. The number of probes targeting each pathogen in the custom probe libraries. In addition to the pathogens specifically targeted in the samples included in this study (bold font), additional pathogens were included in the probe library designs for pathogen discovery purposes and for future applications of this assay (normal font). Probes libraries included partial-genome (1) or full-genome sequences (2).
