# Supplementary material for: Targeted Enrichment for Pathogen Detection and Characterization in Three Felid Species
Source: J Clin Microbiol. 2017 May 23;55(6):1658–70. doi: 10.1128/JCM.01463-16 (PMC5442522; doi:10.1128/JCM.01463-16)
Supplement: Supplemental material [file JCM.01463-16_zjm999095505s2.pdf]

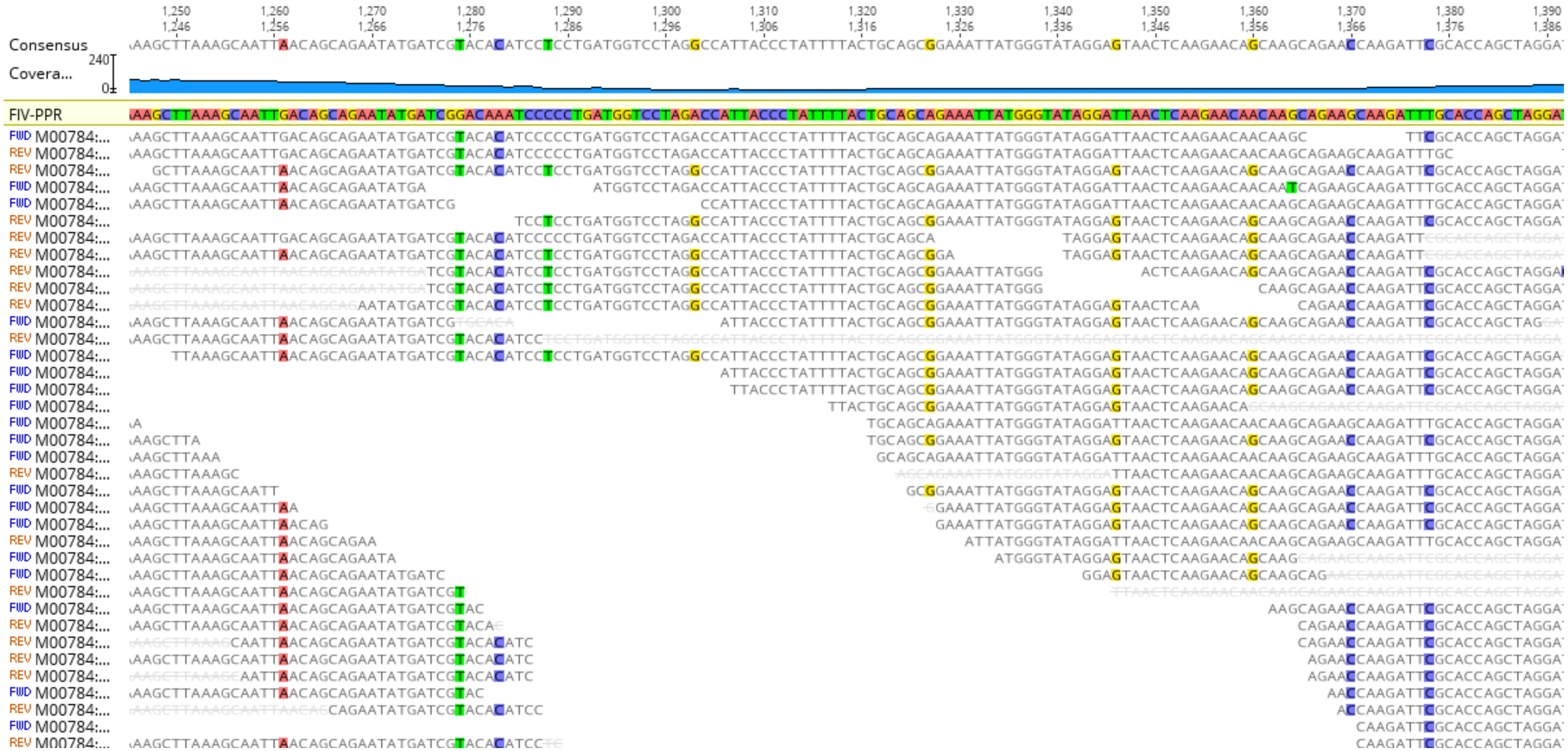

Figure S1: Reads mapped to FIVA demonstrate intra-host diversity within Sample 13. Single-nucleotide polymorphisms (SNPs) that differ from the reference sequence (FIV-PPR) are highlighted with color shading. Intra-host diversity is evident as two bases are present at most of the SNP sites in this portion of the alignment. While not a primary focus of this study, the ability to characterize intra-host diversity is an additional advantage of TGC-NGS over traditional diagnostic assays such as PCR or real time PCR.
